# Supplementary material for: A Humanized Yeast Phenomic Model of Deoxycytidine Kinase to Predict Genetic Buffering of Nucleoside Analog Cytotoxicity
Source: Genes (Basel). 2019 Sep 30;10(10):770. doi: 10.3390/genes10100770 (PMC6826991; doi:10.3390/genes10100770)
Supplement: Supplementary file 1 [file genes-10-00770-s001.zip › Additional_Files/Additional_File7_GOTermHeatmaps.zip!/Additional_File7_GOTermHeatmaps/U2-type_spliceosomal_complex.pdf]

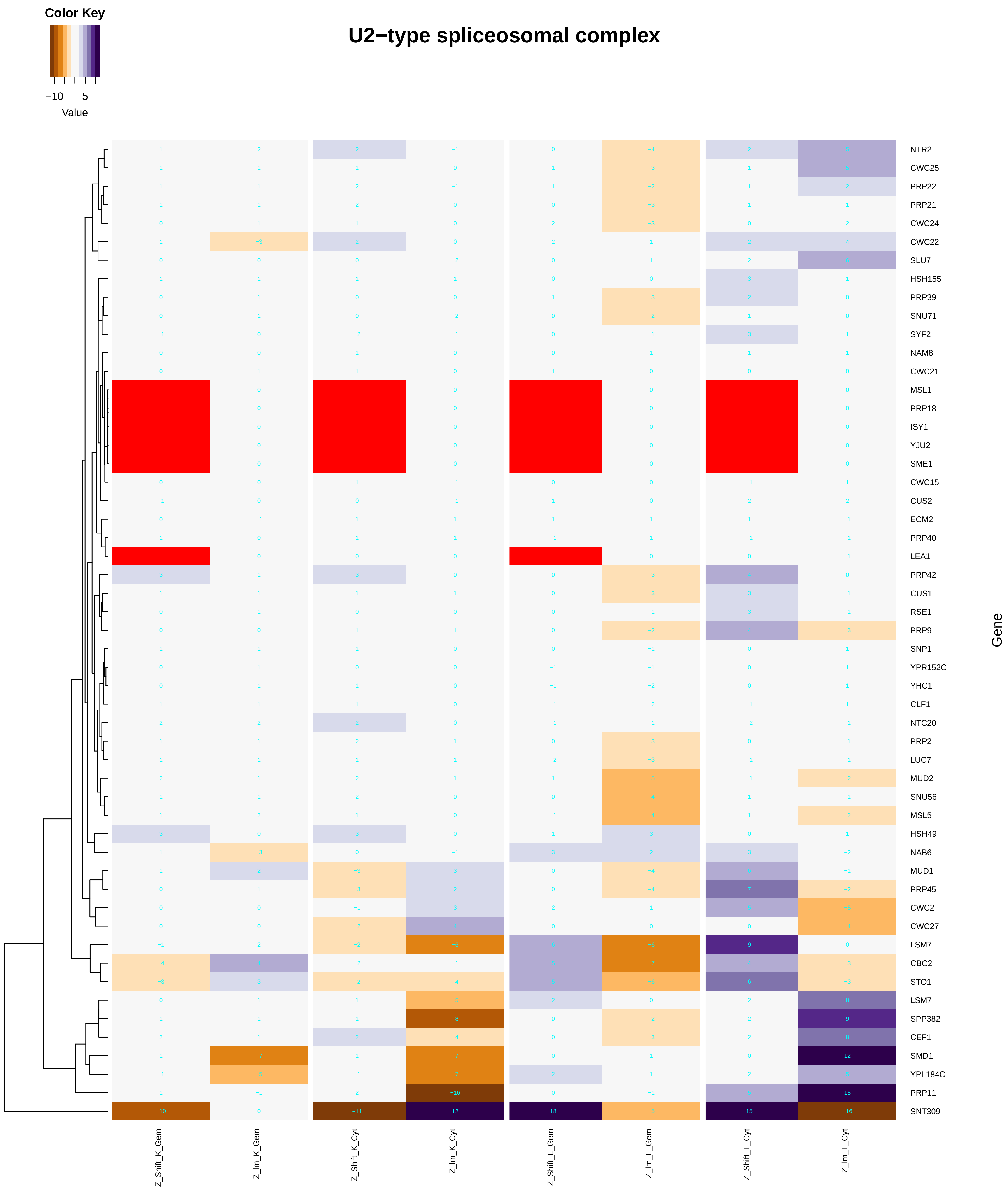

U2−type spliceosomal complex
Color Key
−10
5
Value
NTR2
CWC25
PRP22
PRP21
CWC24
CWC22
SLU7
HSH155
PRP39
SNU71
SYF2
NAM8
CWC21
MSL1
PRP18
ISY1
YJU2
SME1
CWC15
CUS2
ECM2
PRP40
LEA1
PRP42
CUS1
RSE1
PRP9
SNP1
YPR152C
YHC1
CLF1
NTC20
PRP2
LUC7
MUD2
SNU56
MSL5
HSH49
NAB6
MUD1
PRP45
CWC2
CWC27
LSM7
CBC2
STO1
LSM7
SPP382
CEF1
SMD1
YPL184C
PRP11
SNT309
Gene
Z_Shift_K_Gem
Z_Im_K_Gem
Z_Shift_K_Cyt
Z_Im_K_Cyt
Z_Shift_L_Gem
Z_Im_L_Gem
Z_Shift_L_Cyt
Z_Im_L_Cyt

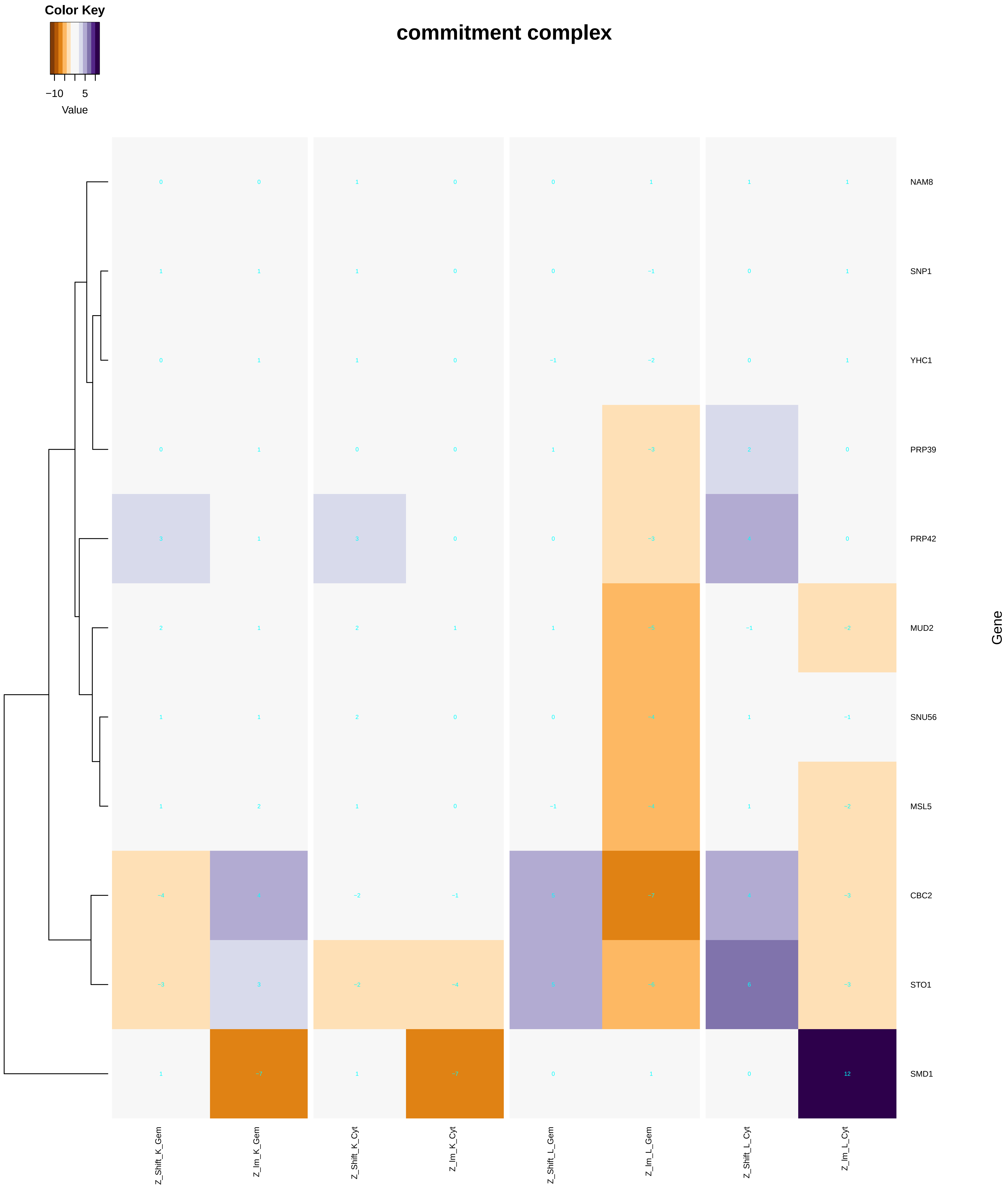

Color Key
−10
5
Value
commitment complex
NAM8
SNP1
YHC1
PRP39
PRP42
MUD2
SNU56
MSL5
CBC2
STO1
SMD1
Gene
Z_Shift_K_Gem
Z_Im_K_Gem
Z_Shift_K_Cyt
Z_Im_K_Cyt
Z_Shift_L_Gem
Z_Im_L_Gem
Z_Shift_L_Cyt
Z_Im_L_Cyt

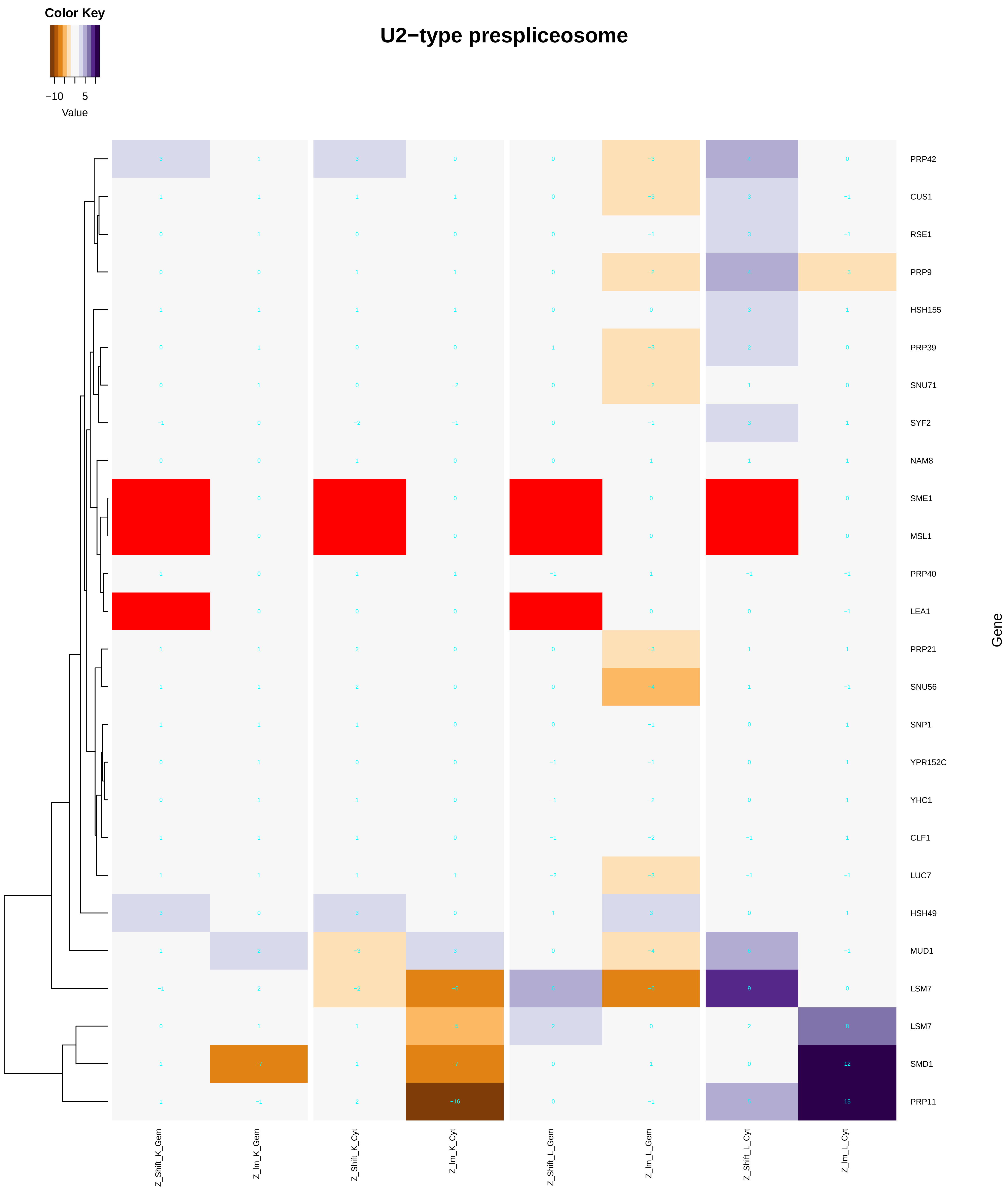

Color Key
−10
5
Value
U2−type prespliceosome
PRP42
CUS1
RSE1
PRP9
HSH155
PRP39
SNU71
SYF2
NAM8
SME1
MSL1
PRP40
LEA1
PRP21
SNU56
SNP1
YPR152C
YHC1
CLF1
LUC7
HSH49
MUD1
LSM7
LSM7
SMD1
PRP11
Gene
Z_Shift_K_Gem
Z_Im_K_Gem
Z_Shift_K_Cyt
Z_Im_K_Cyt
Z_Shift_L_Gem
Z_Im_L_Gem
Z_Shift_L_Cyt
Z_Im_L_Cyt

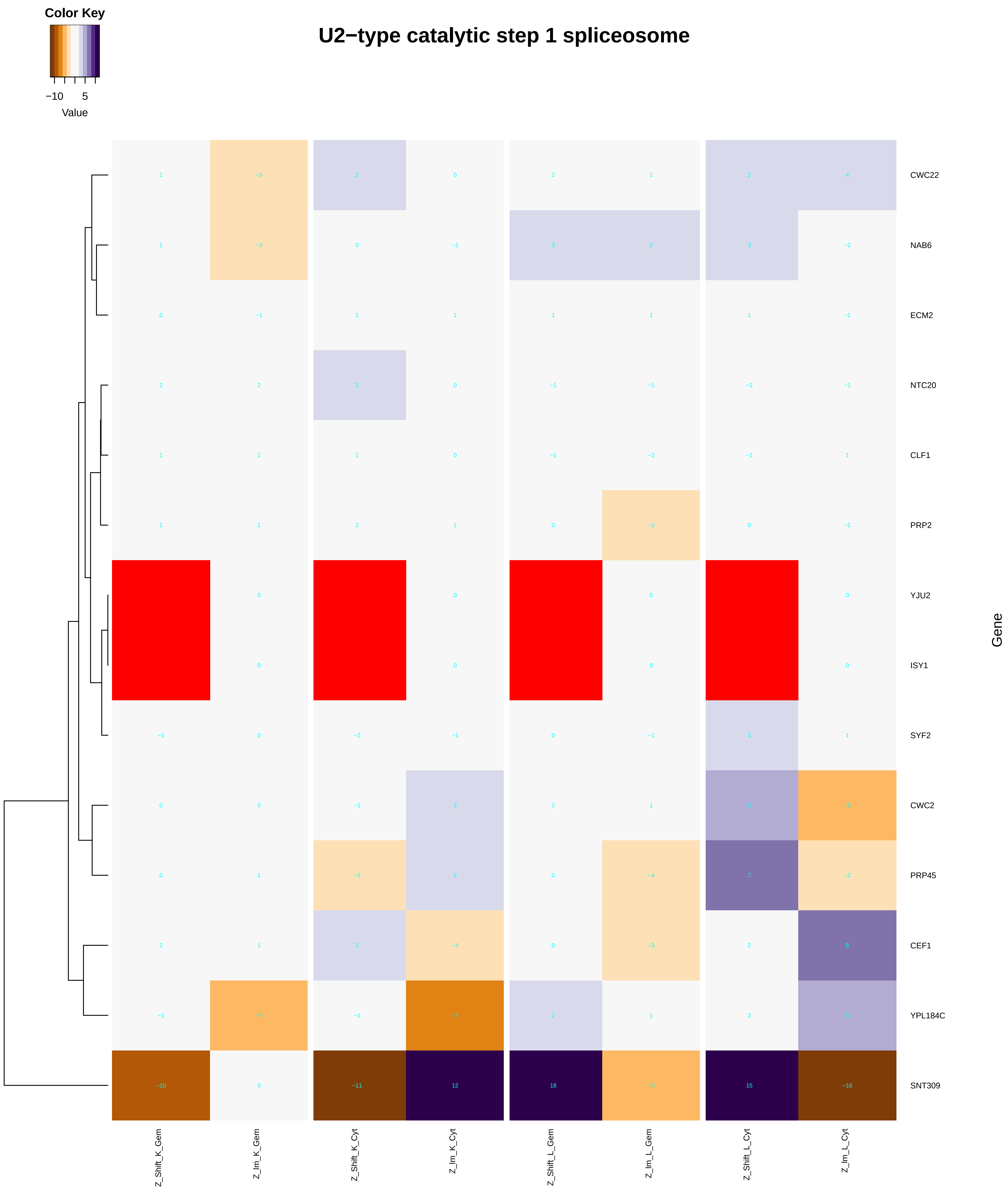
U2−type catalytic step 1 spliceosome
Color Key
−10
5
Value
CWC22
NAB6
ECM2
NTC20
CLF1
PRP2
YJU2
ISY1
SYF2
CWC2
PRP45
CEF1
YPL184C
SNT309
Gene
Z_Shift_K_Gem
Z_Im_K_Gem
Z_Shift_K_Cyt
Z_Im_K_Cyt
Z_Shift_L_Gem
Z_Im_L_Gem
Z_Shift_L_Cyt
Z_Im_L_Cyt

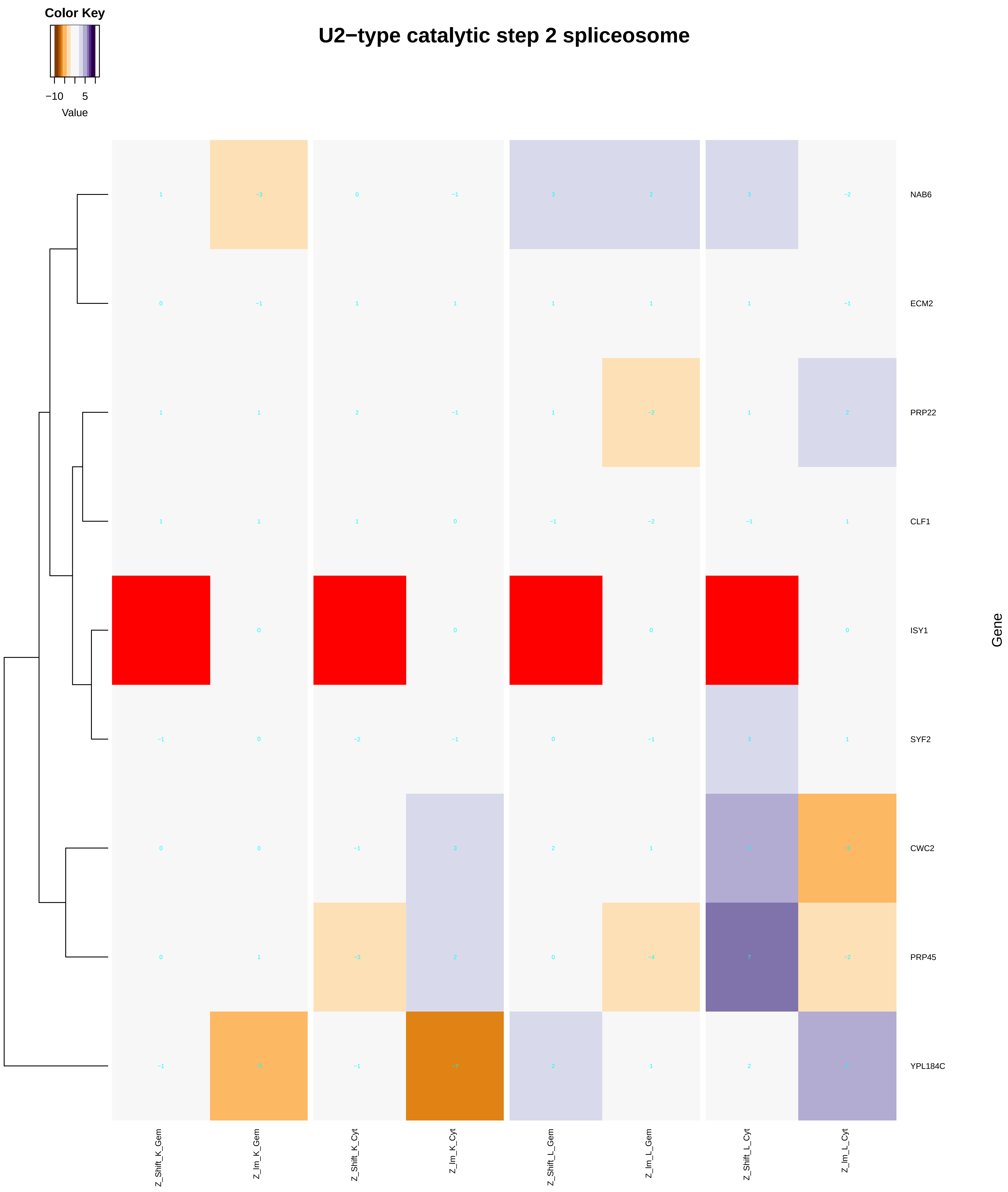
U2−type catalytic step 2 spliceosome
Color Key
−10
5
Value
NAB6
ECM2
PRP22
CLF1
ISY1
SYF2
CWC2
PRP45
YPL184C
Gene
Z_Shift_K_Gem
Z_Im_K_Gem
Z_Shift_K_Cyt
Z_Im_K_Cyt
Z_Shift_L_Gem
Z_Im_L_Gem
Z_Shift_L_Cyt
Z_Im_L_Cyt

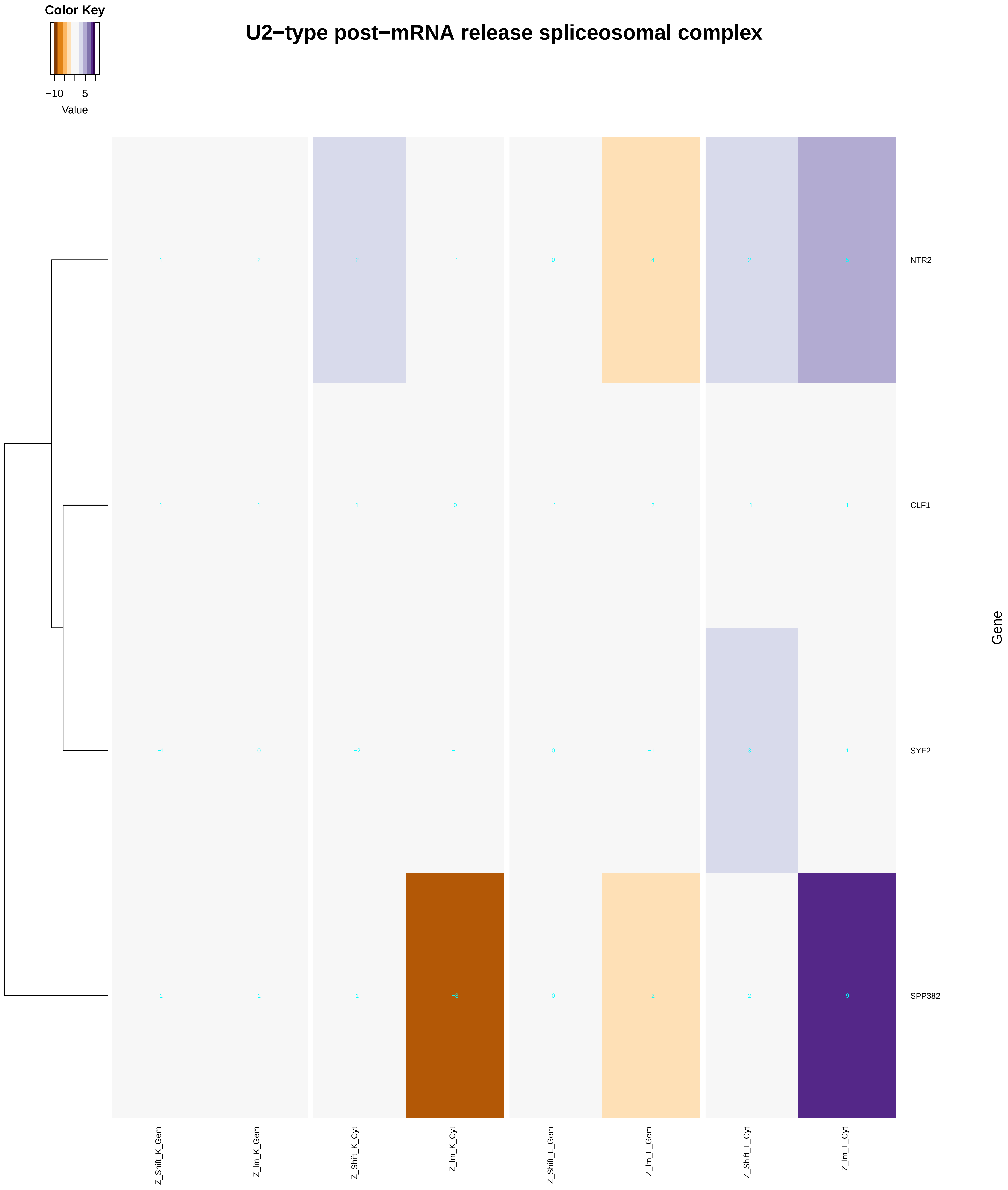
Color Key
−10
5
Value
U2−type post−mRNA release spliceosomal complex
1
2
2
−1
0
−4
2
5
NTR2
1
1
1
0
−1
−2
−1
1
CLF1
−1
0
−2
−1
0
−1
3
1
SYF2
1
1
1
−8
0
−2
2
9
SPP382
Gene
Z_Shift_K_Gem
Z_Im_K_Gem
Z_Shift_K_Cyt
Z_Im_K_Cyt
Z_Shift_L_Gem
Z_Im_L_Gem
Z_Shift_L_Cyt
Z_Im_L_Cyt

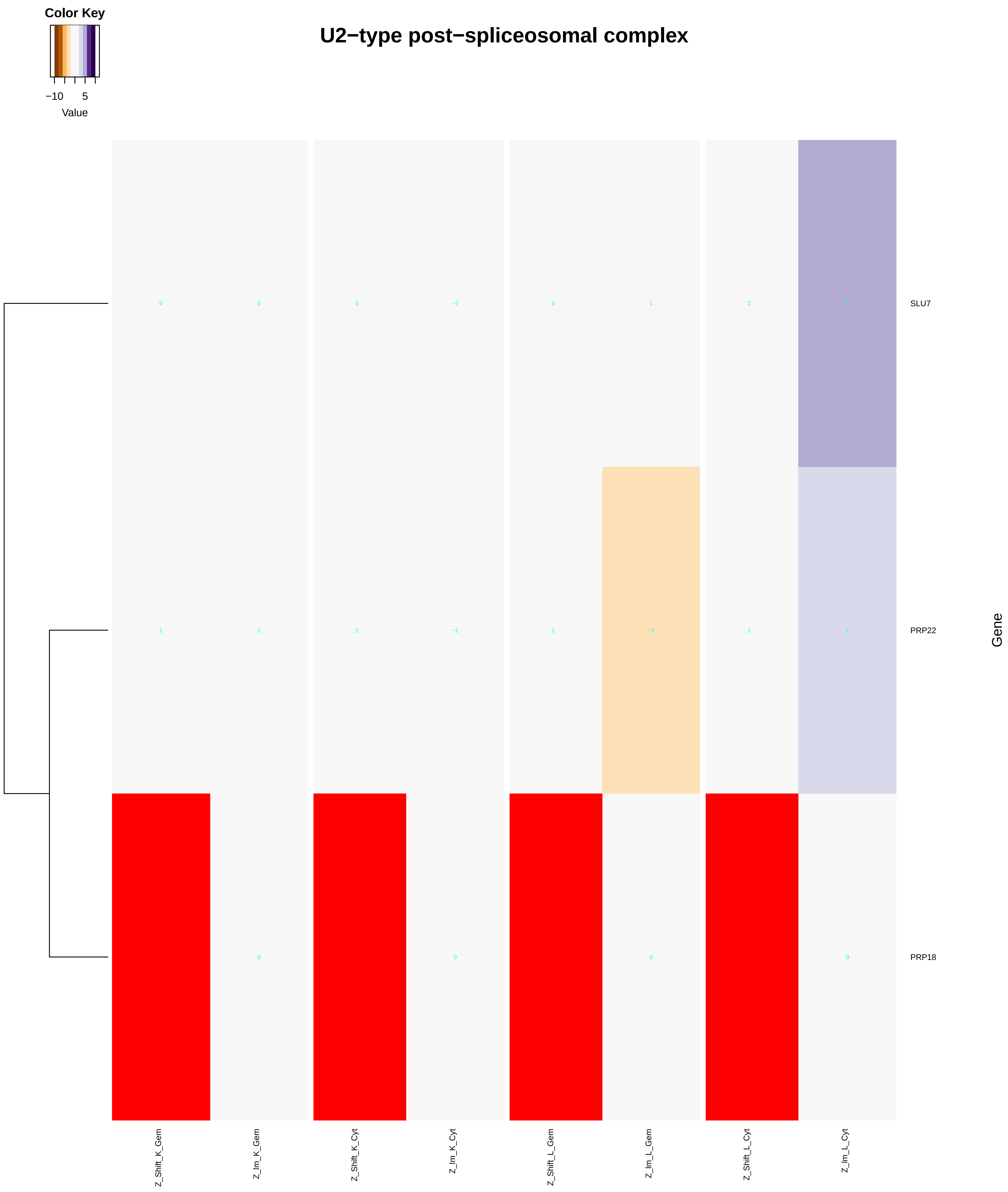

U2−type post−spliceosomal complex
Color Key
−10
5
Value
Gene
SLU7
PRP22
PRP18
Z_Shift_K_Gem
Z_Im_K_Gem
Z_Shift_K_Cyt
Z_Im_K_Cyt
Z_Shift_L_Gem
Z_Im_L_Gem
Z_Shift_L_Cyt
Z_Im_L_Cyt
